# Ecological Risk Assessment and Source Identification of Heavy Metals in Soils from Shiyang River Watershed in Northwest China

**DOI:** 10.3390/toxics11100825

**Published:** 2023-09-29

**Authors:** Jie Liao, Tao Wang, Jianhua Gui, Hengping Zhang, Cuihua Huang, Xiang Song, Shengyin Zhang

**Affiliations:** 1 Northwest Institute of Eco-Environment and Resources, Chinese Academy of Sciences, Lanzhou 730000, China; wt@lzb.ac.cn (T.W.); hch@lzb.ac.cn (C.H.); songxiang@lzb.ac.cn (X.S.); zhangseepage@126.com (S.Z.); 2 Gansu Salinization Field Observation and Research Station, Lanzhou 730000, China; 3 Gulang County Agricultural and Rural Bureau, Wuwei 733199, China; guijh1798@sina.com; 4 Gansu Qilian Mountain National Nature Reserve Management and Protection Center Haxi Nature Reserve Station, Wuwei 733200, China

**Keywords:** heavy metal, biological toxicity, ecological risks, source identification, Shiyang River Watershed

## Abstract

Shiyang River Watershed is an important ecological barrier and agricultural production area in Northwest China, and the study of soil heavy metal content, distribution, and sources is important for agricultural product safety, pollution control, and ecosystem health. In this paper, 140 soil samples were collected from 28 stations to assess the level of heavy metal (Arsenic (As), Copper (Cu), Lead (Pb), Cadmium (Cd), Chromium (Cr), Mercury (Hg), Nickel (Ni), Zinc (Zn)) contamination, pollutant sources and influencing factors of soil in Shiyang River Watershed through determination of the metal contents and statistical analysis. The results indicated that the soils in the study area are typical saline soils in arid zones. The enrichment factors (*EF*) of As, Cr, Cu, Ni, Zn, and Pb indicate no contamination, and the EFs of Cd and Hg suggested minor contamination. Although the concentrations of Cd and Hg in soil are lower than others, they are more biotoxic and exhibit a moderate–high ecological risk. The index of geoaccumulation (*I_geo_*) values reflect that most of the stations, especially the three groups of samples from depths of 10–20 cm, 20–40 cm, and 40–80 cm, are below the contamination threshold for all heavy metals. The chemical speciation of heavy metals, principal component analysis, and correlation analysis showed that Cr, Cu, Pb, Cd, Ni, and Zn mainly come from the natural accumulation upon weathering of soil-forming matrices. Hg and As mainly come from anthropogenic contributions. The effect of agricultural crop cultivation on soil heavy metal contamination is mainly through farm irrigation and crop–soil interactions, which accelerate the release of heavy metals through the weathering of soil-forming parent material and irrigation, which transports the heavy metals below the surface. The results of this study can provide a scientific basis for the involved authorities to formulate reasonable policies on environmental protection and pollution control.

## 1. Introduction

Soil heavy metal contamination is a growing concern worldwide due to its potential negative impacts on human health, the environment, and agriculture [1,2,3,4,5,6,7,8]. Heavy metals are naturally occurring elements, but their concentration in soil has increased significantly due to human activities such as industrialization, mining, and agriculture [9,10,11,12]. Several factors contribute to the presence of heavy metals in soils, including natural processes, human activities, and industrial emissions. Natural factors such as the weathering of rocks, volcanic eruptions, and soil erosion can release heavy metals into the soil [13,14,15,16]. Human activities such as mining, industrial activities, and the use of fertilizers and pesticides can also contaminate soils with heavy metals [17,18,19,20,21]. Additionally, atmospheric deposition of heavy metals from air pollution is another significant factor [22,23]. The concentration of heavy metals in soil is influenced by several factors such as soil pH, organic matter content, soil texture, and redox potential [24,25,26,27]. Understanding the factors that affect the presence of heavy metals in soils is crucial for developing effective strategies to mitigate contamination and protect human health and the environment.

The Shiyang River Watershed, situated in the eastern part of the Qilian Mountains in Gansu Province, is a crucial agricultural region that plays a significant role in the country’s food production. Covering an area of approximately 22,000 square kilometers and home to over 2 million people, the Watershed primarily relies on agriculture for livelihood. Wheat, maize, and potatoes are the main crops grown in the region. However, the problem of water shortage has become increasingly serious over the past decade, despite rapid economic and social development in Wuwei City, located within the watershed. Agricultural land in the area is affected by various factors, including desertification, loess erosion, agro-biology, industrial emissions and salinization [28,29,30,31]. The concentrations of As, Cd, Co, Cr, Cu, Hg, Ni, Pb, and Zn in agricultural soils around Wuwei City were measured by ICP-MS. Also, the enrichment factors (*EF*), Index of Geo-Accumulation (*I_geo_*), Potential Ecological Risk Index (*RI*), and Risk Assessment Code (RAC) were calculated. The heavy metals and ions were investigated by principal component analysis to understand the heavy metal sources and influencing factors. This paper presents the ecological risk assessment and source identification of heavy metals in soil samples collected from cultivated, abandoned, and newly cultivated land. It could provide a scientific basis for the prevention and control of potential contamination of cropland soils in the Shiyang River Watershed.

## 2. Materials and Methods

### 2.1. Study Area and Data Collection

The Shiyang River Watershed is located in the eastern part of the Heshi area of Gansu Province, at the northern foot of the Qilian Mountains, between the Badanjilin Desert and Tengger Desert [32,33]. The geographic coordinates are 101°22’ –104°04’ east longitude and 37°07’ –39°27’ north latitude. The administrative division mainly includes Liangzhou District, Minqin County, and Gulang County of Wuwei City. The oases in the Shiyang River Watershed are mainly distributed in the middle and lower reaches. The warm and wet zone is located in the middle reaches of the watershed, with an altitude of 1500–2000 m, annual precipitation of 150–300 mm, and annual evaporation of 1300–2000 mm. The downstream warm arid zone includes all of Minqin, northern Gulang, and northeastern Liangzhou, with an elevation of 1300–1500 m, annual precipitation less than 150 mm, and annual evaporation of 2000–2600 mm in the northern part of Minqin, which is close to the fringe of the Tengger Desert.

**Figure 1:**
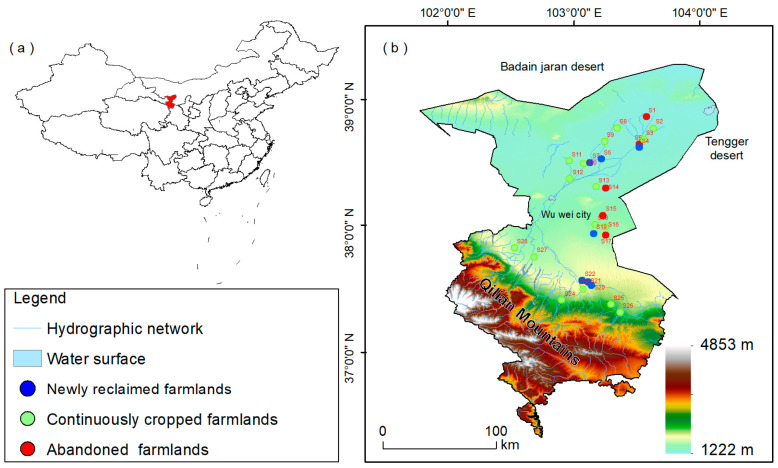
Location of the study area (**a**) and sample sites (**b**).

A total of 140 soil samples were collected in September–November 2022, involving 28 stations, mainly in Liangzhou District, Minqin County, and Gulang County of Wuwei City (Figure 1). Each station was sampled at 0–3, 3–10, 10–20, 20–40, and 40–80 cm depths for a total of 5 samples. According to the current status of land cultivation, the 28 stations will be categorized into three types: newly reclaimed farmlands for land cultivated and planted after 2020, continuously cropped farmlands for land cultivated and planted since 2000, and abandoned farmlands for land cultivated before 1990, which was forced to abandon cultivation since 2000 as a result of insufficient water supply.

### 2.2. Chemical Analysis

#### 2.2.1. pH, Electrical Conductivity (EC), Anions, and Cations

All soil samples were dried and finely ground to 18 mesh for indoor analysis. Five grams (g) of air-dried soil sample were placed in a 50 mL flask, mixed with 25 mL of distilled water, shaken for 5 min and left to stand for 10 min. The pH and conductivity of the suspension were determined using a pH meter (PB−10) [34] and a conductivity meter (DDSJ−308A) [35,36], respectively. Ion chromatography (Metrohm, Zofingen, Switzerland) was used for the determination of Cl^−^, ${\mathrm{SO}}_{4}^{2-}$, ${\mathrm{NO}}_{3}^{-}$, K^+^, Na^+^, Ca^2+^, and Mg^2+^. ${\mathrm{CO}}_{3}^{2-}$ and ${\mathrm{HCO}}_{3}^{-}$ were determined by double indicator-neutralization titration [37].

#### 2.2.2. Major Elements, Trace Elements Measurements

The soil samples were dried in an oven at 55 °C, the obvious foreign matter was removed, and the remaining part was ground and sieved (100 mesh, 150 μm), then the samples were mixed homogeneously and stored in a self-sealing bag. A certain amount of the sample was weighed and put into a digestion tube, where the sample underwent microwave digestion with nitric acid–hydrofluoric acid–perchloric acid (HF-HNO_3_-HClO_4_). The acid was heated to 150 °C on an electric heating plate, and then the sample was cooled down to room temperature, brought to 50 mL volume and then filtered through a 0.45 μm membrane to be stored for measurement [38]. Major elements, e.g., aluminum (Al), titanium (Ti), etc., were determined using an inductively coupled plasma emission spectrometer (PerkinElmer Optima 8000, Waltham, MA, USA) and trace elements were determined using an inductively coupled plasma mass spectrometer (ICP-MS, Agilent 8900, Palo Alto, CA, USA). Atomic fluorescence spectrometry (AFS) was used to determine the contents of Hg and As in the digestion solution.

#### 2.2.3. Sequential Extraction Procedure (BCR)

To analyze soil heavy metal forms, 28 surface soil samples were chosen and weighed accurately to 1.00 g in 100 mL polypropylene centrifuge tubes. A modified BCR procedure was used for the analysis. For the exchangeable fraction (F1), 30 mL of 0.11 mol/L CH_3_COOH was used; for the reducible fraction (F2), 30 mL of 0.1 mol/L NH_2_OH-HCl was used; for the oxidizable fraction (F3), 5 mL of 8.8 mol/L H_2_O_2_ was followed by 25 mL of 1 mol/L CHCOONH_4_; for the residual fraction (F4), HNO_3_-HF-HClO_4_ was used [39]. The extracts and digests from the above steps were used to determine Cd, Co, Cr, Cu, Ni, Pb, and Zn by ICP-MS (ICP-MS, Agilent 8900, Palo Alto, CA, USA), while As and Hg were determined using the atomic fluorescence spectrometer AFS−8220.

#### 2.2.4. Quality Control (QC)

Soil soluble ions (ion chromatography, Metrohm, Switzerland) were detected with a limit of detection of 1 mg/kg and an error of detection of ±5%. ICP-OES and ICP-MS were used for elemental measurements using a standard reference sample (GSS−18), blanks, and replicate samples in order to control the quality of the whole analytical process. The reproducibility of the elements in the samples was greater than 95% and the recoveries of the standard samples ranged from 90% to 105% (Table A1, Appendix A).

### 2.3. Ecological Risk Assessment

The enrichment factor (*EF*) [40,41,42], geological accumulation index (*I_geo_*) [43,44], potential ecological risk index (*RI*) [45,46,47] and risk assessment index (RAC) [48] were used to assess the ecological risk and source analysis of soil heavy metals. The formulas for these parameters can be found in reference [1,2,3,4,5,6,7,8].

The background element values were obtained from the samples at station S21 (Table 1), which is located near the newly cultivated land. It is a soil from the Tengger Desert, and has very little anthropogenic influence.

### 2.4. Statistical Analysis

Correlation analysis and principal component analysis (PCA) were carried out using Origin 2022 (OriginLab Inc., Northampton, MA, USA). Maps of the sampling sites (Figure 1) were produced using ArcGIS 10.2.

## 3. Results and Discussions

### 3.1. Physicochemical Properties of Soils

**Figure 2:**
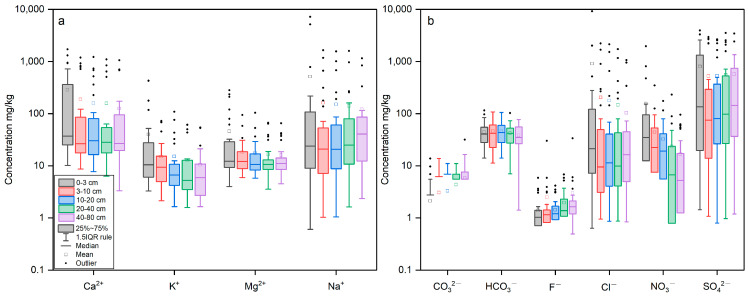
Box plot of anions (**a**) and cations (**b**) for the soil samples from the Shiyang River Watershed.

Figure 2 and Table A2 illustrate the EC, pH values, and the concentrations of Ca^2+^, K^+^, Mg^2+^, Na^+^, ${\mathrm{CO}}_{3}^{2-}$, ${\mathrm{HCO}}_{3}^{-}$, Cl^−^, ${\mathrm{NO}}_{3}^{-}$, and ${\mathrm{SO}}_{4}^{2-}$ extracted from soil samples collected across five strata within the study area. Over the layers ranging from the first to the fifth, the EC values exhibit mean values of 2654, 1148, 1103, 1045, and 896 mS/cm. Similarly, the pH mean values were 8.26, 8.45, 8.48, 8.56, and 8.55. In the first stratum, concentrations of Ca^2+^, K^+^, Mg^2+^, and Na^+^ exhibited mean values of 287.0988, 39.880, 45.665, and 511.503 mg/L, respectively. The underlying layers also reveal analogous concentration trends for these cations, each characterized by distinct concentration ranges and corresponding mean values (Figure 2a). Within the initial layer ${\mathrm{CO}}_{3}^{2-}$, ${\mathrm{HCO}}_{3}^{-}$, Cl^−^, ${\mathrm{NO}}_{3}^{-}$, and ${\mathrm{SO}}_{4}^{2-}$ concentrations exhibited mean values of 2.120, 44.209, 908.358, 155.151, and 798.880 mg/L, respectively (Figure 2b). In summary, the measured soil parameters of the soil in the study area were pH > 7, EC ranging from 0.047 to 24.7, with cation contents in the following order: Na^+^ > Ca^2+^ > Mg^2+^ > K^+^, and anion contents in the following order: ${\mathrm{SO}}_{4}^{2-}$ > Cl^−^ > ${\mathrm{NO}}_{3}^{-}$ > ${\mathrm{HCO}}_{3}^{-}$ > ${\mathrm{CO}}_{3}^{2-}$. Moreover, the formation of surface efflorescent crusts of soluble salts was evident, which was caused by salt transport to the surface during capillary evaporation. The above results reflect the fact that the study soil is an arid zone saline soil.

### 3.2. The Concentration of Heavy Metals in Soils

**Figure 3:**
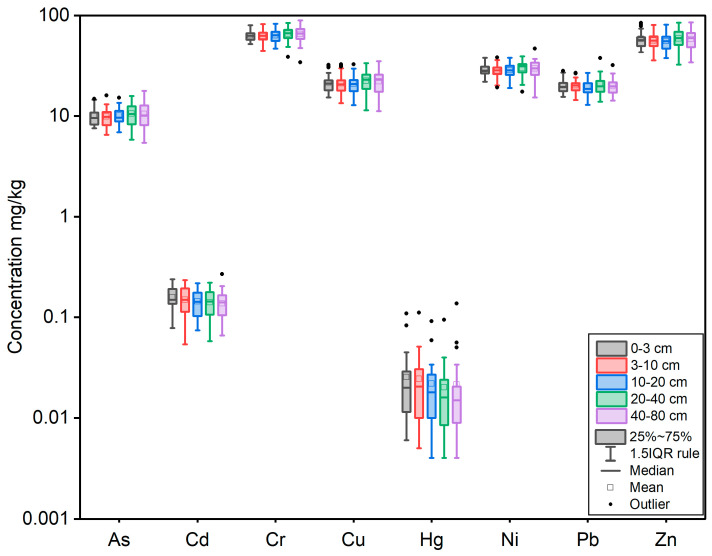
Box plot of heavy metal concentrations for the soil samples from the Shiyang River Watershed.

Figure 3 and Table A3 provide the concentrations of heavy metals such as As, Cd, Cr, Cu, Hg, Ni, Pb, and Zn in soil samples in the present study. The mean values of these heavy metals for all samples were in the following order: Cr > Zn > Ni > Cu > Pb > As > Cd > Hg. In the first layer, concentrations of As, Cd, Cr, Cu, Hg, Ni, Pb, and Zn exhibited mean values of 9.99, 0.16, 63.70, 21.72, 0.03, 29.24, 20.19, and 58.94 mg/kg, respectively. In the second layer, concentrations of As, Cd, Cr, Cu, Hg, Ni, Pb, and Zn exhibited mean values of 9.94, 0.15, 62.97, 21.35, 0.03, 28.55, 20.13, and 56.97 mg/kg, respectively. The heavy metal contents of the samples from these three layers were similar to the upper two layers. However, the median statistics showed that the heavy metal contents tended to increase with depth, except for Cd and Hg.

### 3.3. Geochemical Fractionations of Heavy Metals

**Figure 4:**
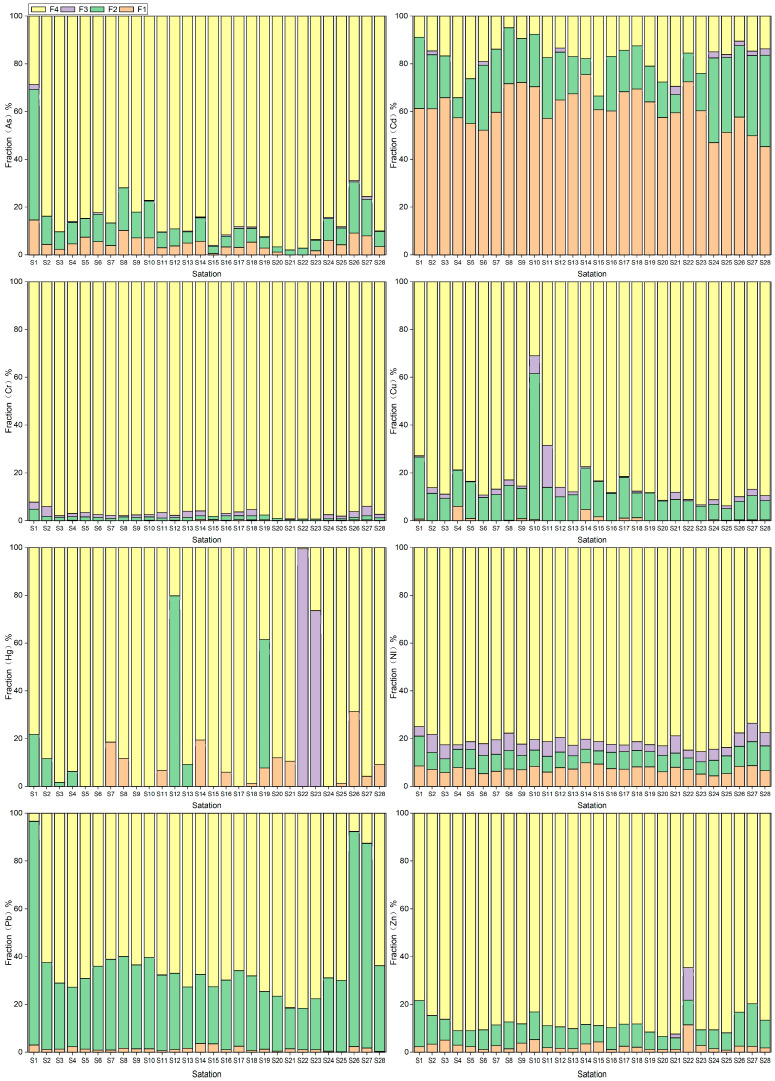
Percentage distribution of heavy metals: the exchangeable fraction (F1), the reducible fraction (F2), the oxidizable fraction (F3), and residual fraction (F4) for the surface soil samples from the 28 stations.

The BCR extraction method is a useful tool for classifying soil heavy metals into four distinct chemical forms. These forms include acid extractable, reducible (Fe-Mn oxide bound), oxidizable (organic bound), and residue. The acid extractable form encompasses the highly mobile exchangeable and carbonate bound forms, which can be readily absorbed and utilized by organisms. On the other hand, heavy metals in the residue form are primarily found in the crystalline matrix of primary and secondary minerals like silicates. These minerals are quite stable and are not easily released under normal natural conditions. The chemical speciation of heavy metals in soil is closely related to the study of heavy metals and migration, transformation, cycling processes and environmental impacts. Chemical speciation analysis of heavy metals is more critical than the total soil content. This helps to accurately assess the environmental impact of soil heavy metals. The heavy metal composition of newly reclaimed, continuously cropped, and abandoned farmlands has been analyzed in this study. The results indicate that, except for Cd and Pb, the residual fractions of As, Cr, Cu, Hg, Ni, and Zn are predominantly hosted by scarcely soluble minerals (Figure 4). This implies limited mobility and uptake by plants. The oxidizable fractions of these heavy metals are generally low, while the reducible fractions of Cd and Pb are relatively higher. The acid extractable fraction of Cd is relatively high in all three types of farmlands. These findings suggest that proper measures should be taken into consideration with respect to preventing heavy metal contamination in agricultural lands.

### 3.4. Assessment of Potential Ecological Risk

#### 3.4.1. *EF* and *I_geo_*

**Figure 5:**
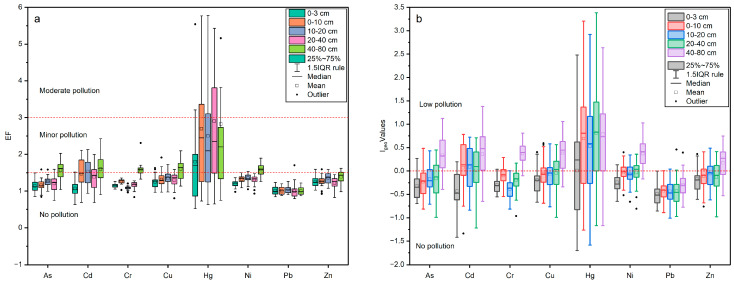
*EF* (**a**) and *I_geo_* (**b**) values of heavy metals for the soil samples from the Shiyang River Watershed.

The *EF* mean value of the samples showed the following order: Hg (1.86) > Cu (1.35) > Ni (1.34) > Cd (1.33) > Zn (1.27) > Cr (1.24) > As (1.23) > Pb (1.00). The *EF* of all the heavy metals was less than 2 in the Shiyang River Watershed, indicating that the detected heavy metals do not pose any remarkable environmental trouble (Figure 5a). The average concentration of the *EF* < 1.5 indicates that the heavy metals are entirely derived from natural sources. The *EF* values of As, Cr, Cu, Ni, Zn, and Pb were less than 1.5, suggesting no contamination, and the EFs of Cd and Hg have values between 1.5 and 3, i.e., contamination is minor. Some samples had *EF* values for Hg exceeding 3, indicating moderate contamination. However, all values are below the risk screening values established by Chinese regulations [1,2,3,4,5,6,7,8]. All the heavy metal elements except Pb showed low *EF* values for surface samples and high *EF* values for deeper samples. This suggests that heavy elements in arid areas are enriched in the lower (tillage) layer, contrary to the surface transport effect as in the case of soluble salts.

The *I_geo_* values for As (mean, 0.28), Cr (0.33), Cu (0.34), Ni (0.34), and Zn (0.13) in the samples showed low contamination. Overall, the *I_geo_* mean value of these samples showed the following order: Hg (0.28) > Cu (−0.06) > Ni (−0.05) > Cd (−0.09) > Zn (−0.13) > As (−0.16) > Cr (−0.17) > Pb (−0.47) (Figure 5b). As with the *EF*, the lowest *I_geo_* values were found for the most superficial samples at all stations.

#### 3.4.2. Potential Ecological Risk Index

**Figure 6:**
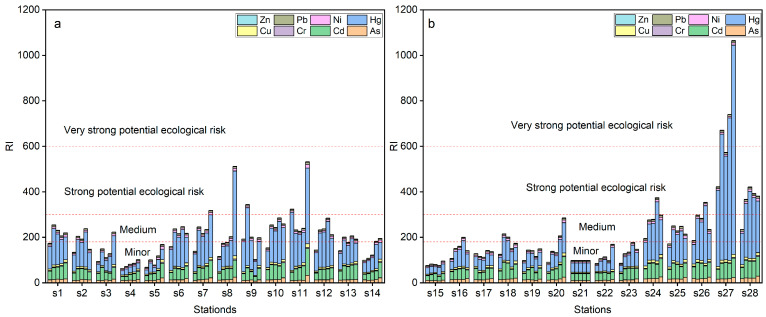
*RI* values of heavy metals for the S1–S14 (**a**) and S15–S28 (**b**) from the Shiyang River Watershed.

**Figure 7:**
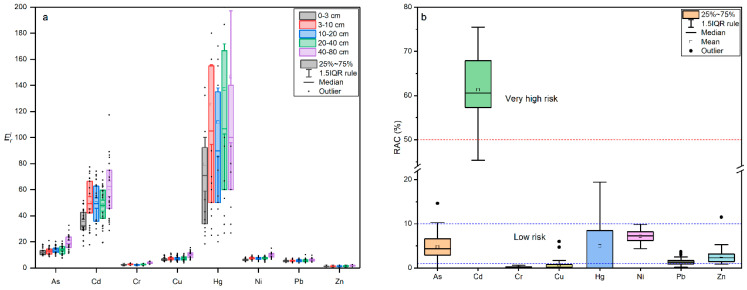
$E_{r}^{i}$ (**a**) and RAC (**b**) values of heavy metals for the soil samples from the Shiyang River Watershed.

The ecological risk of heavy metal contaminants in 140 soil samples was evaluated by calculating the sum of eight potential risk coefficients. The results showed that the majority of samples exhibited a medium level of ecological risk, with only 3 samples from site S27 displaying a very high risk and 16 samples showing high risk. Two samples were found to have a low ecological risk. The order of potential ecological risk for individual heavy metals in the study area was Hg > Cd > As > Cu > Ni > Pb > Cr > Zn, with Hg and Cd posing a moderate risk and As, Ni, Cu, Cr, Pb, and Zn posing a slight risk. These findings suggest that most heavy metals in the soil had a low ecological risk. These results are presented in Figure 6 and Figure 7a.

#### 3.4.3. Risk Assessment Code

Risk Assessment Codes (RACs) for heavy metals in topsoil samples from 28 sites are presented in Figure 7b and indicate that heavy metals such as As, Cr, Cu, Hg, Ni, Pb and Zn pose low or no risk to the environment due to low mobility and bioavailability (RAC < 10%). Cadmium, however, is considered to be a high risk to arable soils (RAC > 60%). It is worth noting that different approaches to estimating the ecological risk of heavy metals can lead to inconsistent results, mainly due to different methods of assessing contamination risk. For example, *EF* and *I_geo_* focus on the enrichment and accumulation of total soil heavy metals relative to soil background values, while RI assesses ecological risk based on the biotoxicity of different heavy metals. RAC emphasizes the mobility of heavy metals, i.e., their ability to be taken up by plants. In general, most of the soils in the study area had low levels of heavy metals and were uncontaminated or lightly contaminated. In conclusion, the RAC results suggest that most soils in the study area are not significantly contaminated with heavy metals, with the exception of Hg and Cd. However, the levels of Hg and Cd in the soils of the study area are much lower than the national and international standards for soil contamination [1,2,3,4,5,6,7,8]. These two heavy metals are of negligible biotoxicity in the soils of the Shiyang River Basin.

## 4. Discussions

### 4.1. Source Identification of Heavy Metals

**Figure 8:**
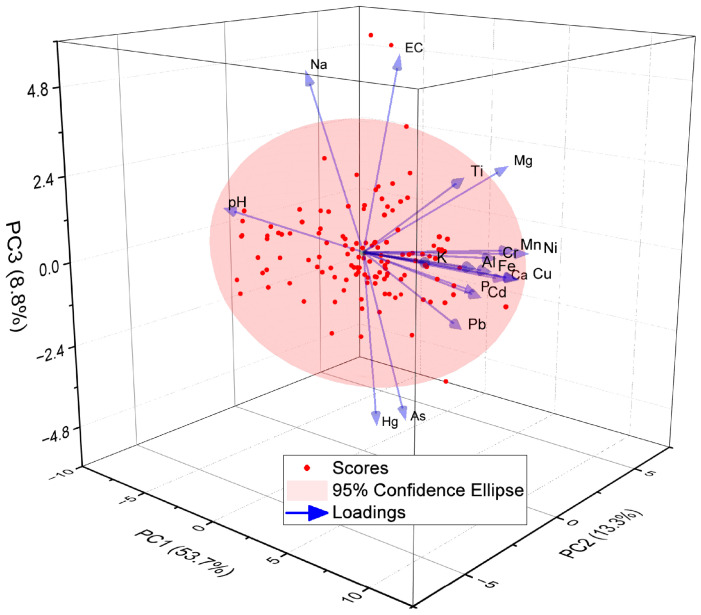
PCA for the eight heavy metals, pH, EC, and elements in the soil samples from the Shiyang River Watershed.

In this study, principal component analysis (PCA) and correlation analysis were used to determine the sources of heavy metals and their influencing factors. The elements analyzed included As, Cd, Cr, Cu, Hg, Ni, Pb, Zn, pH, EC and other elements (Figure 8). The results of PCA were validated by Bartlett’s test of sphericity (*p* < 0.001), confirming the appropriateness of the PCA method. The first principal component (PC1), second principal component (PC2) and third principal component (PC3) explained 53.7%, 13.3% and 8.8% of the total variance of the soil samples, respectively. PC1 showed positive loadings for Al, Fe, Mn, Cr, Cu, Pb, Cd, Ni, Zn, Ti, and P, suggesting that it may represent migratory accumulation of detrital minerals. PC2 shows significant positive loadings for EC, Ca and Mg and negative loadings for pH. This suggests that PC2 may represent chemical precipitates such as CaCO_3_ and MgCO_3_. PC3 showed significant positive loadings for EC and Na and negative loadings for As and Hg. This suggests a specific source of arsenic and mercury in the samples.

**Figure 9:**
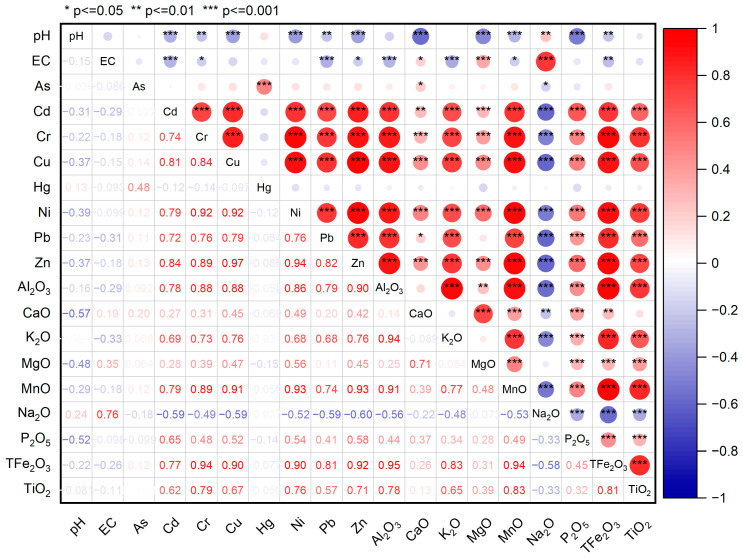
The hot map of correlation between soil heavy metals and influencing factors for the soil samples from the Shiyang River Watershed.

Summarizing, three sources of soil heavy metals in the Shiyang River watershed were identified. The heavy metals Cr, Cu, Pb, Cd, Ni, and Zn have a positive correlation with Al, Mn, Fe and Ti (Figure 9), and are mainly distributed in the soil residue fraction. These heavy metals are a result of natural weathering of soil-forming matrices, particle size sorting during sediment transport, and adsorption by fine-grained clay minerals and organic matter. It is important to note that these heavy metals are not a result of human activities, but rather a natural occurrence. The source of As and Hg in soil appears different from the above, Hg and As mainly come from industrial emissions, transportation pollution sources, and other human activities, and both are affected by the combination of human activities and changes in the natural environment. Wuwei City is an important agricultural and industrial city. Due to the acceleration of industrialization and the use of pesticides and chemical fertilizers, Hg and As enter the soil through atmospheric deposition and migrate with groundwater to irrigation areas.

### 4.2. Factors Affecting Soil Heavy Metals

**Figure 10:**
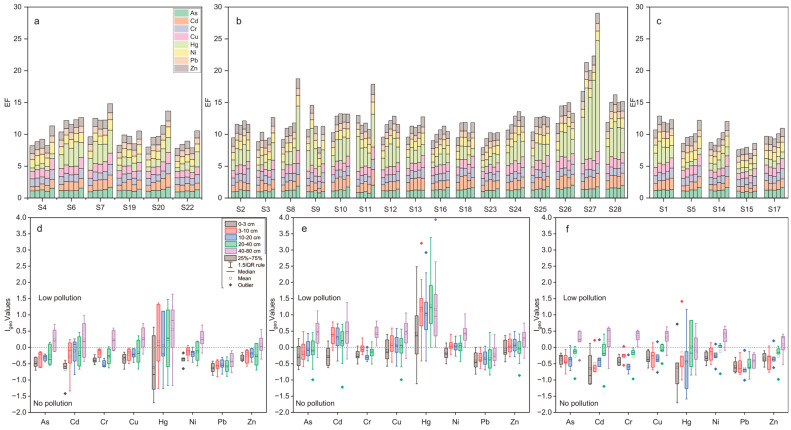
*EF* of Newly reclaimed farmlands (**a**), continuously cropped farmlands (**b**), abandoned farmlands (**c**) and *I_geo_* of newly reclaimed farmlands (**d**), continuously cropped farmlands (**e**), abandoned farmlands (**f**) from the Shiyang River Watershed.

The results of this study have revealed a clear order in the accumulation of soil heavy metals for different years of cultivation. It was found that continuously cropped farmlands have the highest levels of heavy metals, followed by newly reclaimed farmlands and abandoned farmlands (Figure 10). These findings underscore the significant impact that agricultural practices can have on soil quality. The use of fertilizers, pesticides, and irrigation water in farming can all contribute to heavy metal accumulation in soil [20,49,50,51,52,53,54,55]. Fertilizers made from rock phosphate, for example, often contain high levels of cadmium and lead, which can build up over time and cause soil contamination [56,57,58]. Similarly, pesticides may contain heavy metals like mercury and arsenic, which can have long-term effects on soil health [59,60]. Irrigation water is also a common source of heavy metal contamination in soil, particularly from mining and industrial processes [61,62]. Therefore, it is crucial to monitor and manage agricultural practices to minimize the accumulation of heavy metals in soil and prevent further contamination.

Irrigation water in the Shiyang River Basin mainly comes from deep groundwater and a small amount of Yellow River water (purified by sedimentation), while the principal component analysis shows that, except for Hg and As, the source of heavy metals is mainly the weathering of soil-forming substrates. Moreover, heavy metals have a significant cumulative effect in the deep layer (tillage layer). It can be assumed that the impact of agricultural production on soil heavy metals in the study area is mainly manifested by (1) the migration and fixation of heavy metals to deeper depths as a result of prolonged irrigation (drip irrigation), and (2) accelerated weathering and accumulation of heavy metals as a result of the interaction of the soil with the root system of agricultural crops.

## 5. Conclusions

The research shows that the integrated approach, including the modified ecological risk assessment method and biotoxicity assessment method, effectively identifies the potential ecological risk and sources of heavy metals in the soil samples from the Shiyang River Watershed. The results indicated that the soils in the study area are typical saline soils in arid zones. The main cations in the soluble salt of the surface soil are Na^+^ and Ca^2+^, while the anions are Cl^−^ and ${\mathrm{SO}}_{4}^{2-}$. ${\mathrm{CO}}_{3}^{2-}$ and ${\mathrm{HCO}}_{3}^{-}$ (pH > 7). The EFs of As, Cr, Cu, Ni, Zn, and Pb indicate no contamination, whereas the EFs of Cd and Hg point to minor contamination. Some of the soil samples had EF values for Hg exceeding 3, indicating moderate contamination. The *I_geo_* values reflect that most of the stations, especially the three groups of samples from depths of 10–20 cm, 20–40 cm, and 40–80 cm, are low in Cd and Hg, and all other heavy metals are below the contamination levels. Although the concentrations of Cd and Hg in the soil are low, they are more biotoxic and may eventually represent an ecological risk, especially Cd, which is mainly bound to the exchangeable and reducible fraction in the study area.

The chemical speciations of heavy metals, principal component analysis, and correlation analysis showed that Cr, Cu, Pb, Cd, Ni, and Zn mainly come from the natural accumulation through the weathering of soil-forming matrices. Hg and As mainly come from industrial emissions, transportation pollution sources, and other human activities. The effect of agricultural crop cultivation on soil heavy metal contamination in the study area is mainly through farm irrigation and crop–soil interactions, which accelerate the release of heavy metals through the weathering of soil-forming parent material on the one hand, and irrigation on the other hand, which transports the heavy metals below the surface layer. This effect was manifested in the study area as *EF* and *I_geo_* were the highest in old cropland and at 40–80 cm.

## Figures and Tables

**Table 1:** Elemental content of background sample S21.

	Al_2_O_3_ %	As	Cd	Cr	Cu	Hg	Ni	Pb	Zn
		mg/kg							
0–3 cm	9.88	8.09	0.14	52.00	16.20	0.01	22.90	18.70	43.60
3–10 cm	9.36	10.70	0.09	44.60	14.40	0.033	19.20	17.80	40.20
10–20 cm	9.81	11.10	0.09	55.00	14.50	0.01	19.90	17.30	38.40
20–40 cm	9.58	5.44	0.09	50.10	15.10	0.01	20.30	18.20	42.50
40–80 cm	8.71	12.70	0.07	34.00	11.20	0.09	15.30	16.20	34.00

## Data Availability

The data are contained within the article.

## Appendix A

**Table A1:** The results of heavy metals (mg/kg) in standard reference samples (GSS−18).

Sample	GSS−18	GSS−18	GSS−18	GSS−18	GSS−18	Actual Value	Detection Limit (mg/kg)
Cr	54.653	56.212	56.75	53.957	55.721	55 ± 2	0.005
Ni	25.471	24.53	24.686	26.243	25.796	25 ± 1	0.005
Cu	19.981	19.574	29.889	19.532	19.663	19.5 ± 0.5	0.01
Zn	62.627	65.496	63.432	62.653	63.519	63 ± 2	0.02
As	10.289	10.688	11.164	10.667	11.106	10.7 ± 0.5	0.05
Cd	0.146	0.159	0.148	0.158	0.141	0.15 ± 0.01	0.01
Pb	19.985	20.817	19.972	19.858	20.667	20 ± 1	0.005
Hg	0.016	0.014	0.016	0.018	0.013	0.015 ± 0.003	0.005

**Table A2:** Maximum, minimum, and mean values of anions and cations (mg/kg), pH, and EC of soil in Shiyang River Watershed.

		pH	EC	Ca^2+^	K^+^	Mg^2+^	Na^+^	${CO}_{3}^{2-}$	${HCO}_{3}^{-}$	Cl^−^	${NO}_{3}^{-}$	${SO}_{4}^{2-}$
0–3 cm	min	7.58	53.20	10.15	3.28	3.99	0.60	ND.	14.03	0.63	ND.	1.44
	max	8.98	24,700.00	1716.00	428.00	282.00	7196.00	13.80	115.08	12,529.30	1962.92	3952.90
	mean	8.26	2654.03	287.10	39.88	45.67	511.50	2.12	44.21	908.36	155.15	798.88
3–10 cm	min	7.57	47.40	8.68	2.14	5.93	1.03	ND.	11.23	0.96	ND.	1.07
	max	8.96	8010.00	1189.00	72.09	94.46	1642.00	13.80	108.07	2225.00	347.08	2948.53
	mean	8.45	1148.40	186.25	14.97	18.36	161.37	3.06	46.26	204.83	44.00	530.28
10–20 cm	min	7.54	51.60	7.70	1.65	5.78	1.05	ND.	14.03	0.86	ND.	0.80
	max	9.04	7820.00	1221.00	108.70	103.70	1562.00	11.04	105.26	2144.30	175.05	2670.00
	mean	8.48	1103.36	158.19	15.08	17.56	150.82	3.30	45.46	178.53	32.58	534.82
20–40 cm	min	7.74	51.70	6.31	1.58	3.54	1.63	ND.	7.02	0.87	ND.	0.97
	max	9.49	7400.00	1099.00	61.14	66.74	1591.00	11.04	102.45	1727.57	231.63	3523.19
	mean	8.56	1045.14	156.72	12.89	16.55	140.89	4.34	42.35	147.91	23.36	556.97
40–80 cm	min	7.64	49.60	3.34	1.65	4.52	2.35	ND.	1.40	0.84	ND.	1.19
	max	9.64	5850.00	1056.00	53.97	65.23	1150.00	31.75	77.19	1062.46	95.45	3434.88
	mean	8.55	896.10	125.51	10.45	16.03	120.83	5.82	38.09	103.78	16.73	572.41

Note: “ND.” means not detected.

**Table A3:** Maximum, minimum, and mean values (mg/kg) of heavy metals in soil from Shiyang River Watershed.

		As	Cd	Cr	Cu	Hg	Ni	Pb	Zn
0–3 cm	min	7.57	0.08	53.20	15.30	0.01	21.90	15.50	43.00
	max	14.80	0.24	80.10	32.10	0.11	37.70	28.00	84.10
	mean	10.00	0.16	63.70	21.71	0.03	29.24	20.19	58.94
3–10 cm	min	6.51	0.05	45.80	13.40	0.01	20.10	14.40	35.60
	max	16.00	0.24	81.90	32.60	0.11	38.00	26.80	80.30
	mean	9.94	0.15	62.97	21.35	0.03	28.55	20.13	56.97
10–20 cm	min	6.90	0.07	46.90	12.80	ND.	18.90	12.90	37.50
	max	15.20	0.22	82.70	32.50	0.09	37.90	26.70	80.90
	mean	10.19	0.15	63.66	21.13	0.02	28.75	19.52	56.31
20–40 cm	min	5.81	0.06	38.50	11.40	ND.	17.40	13.90	32.30
	max	15.80	0.22	84.50	33.50	0.09	39.10	37.50	85.00
	mean	10.63	0.14	66.53	22.64	0.02	30.14	20.79	59.69
40–80 cm	min	6.21	0.07	47.50	13.30	ND.	20.40	14.20	35.40
	max	17.80	0.27	89.50	35.00	0.14	46.80	31.90	85.60
	mean	10.68	0.14	66.72	22.44	0.02	30.12	20.14	58.89

Note: “ND.” means not detected.
